# Long-term effects following prenatal cocaine exposure: A systematic review

**DOI:** 10.1371/journal.pone.0352587

**Published:** 2026-06-26

**Authors:** Rocco Miazzi, Clara Cestonaro, Francesco Attanasio, Guido Travaini, Cristina Scarpazza, Claudio Terranova

**Affiliations:** 1 Department of Human Neuroscience, Sapienza University of Rome, Rome, Italy; 2 Department of Cardiac, Thoracic, Vascular Sciences and Public Health, University of Padua, Padua, Italy; 3 Department of Clinical Neurosciences, Vita-Salute San Raffaele University, Milan, Italy; 4 Faculty of Medicine, Vita-Salute San Raffaele University, Milan, Italy; 5 Department of General Psychology, University of Padua, Padua, Italy; 6 Laboratory of Translational and Cognitive Neuroimaging, IRCCS San Camillo Hospital, Venice, Italy; Virginia Commonwealth University School of Medicine, UNITED STATES OF AMERICA

## Abstract

Cocaine use represents a global public-health concern, and children’s exposure to this substance is receiving growing attention. Despite the importance of this phenomenon, efforts to isolate cocaine-specific long-term effects are affected by the limited availability of human cohorts followed into adulthood and by the influence of environmental factors and co-exposures. Addressing ongoing debates in the literature, this systematic review synthesizes human evidence on long-term outcomes following prenatal cocaine exposure (PCE), from infancy to early adulthood. A comprehensive search of PubMed and Scopus from inception to August 2025 identified 26 eligible studies. Results suggest that across maturational stages, PCE is consistently associated with early developmental effects (including smaller head circumference and motor delays), deficits in visuospatial, language and executive functions, focal neuroimaging alterations (white-matter microstructure and task-related functional recruitment), growth deficits, and elevated externalizing behaviours. However, evidence is characterised by heterogeneity in exposure assessment, frequent prenatal polysubstance exposure, socioeconomic confounding, caregiving instability and small neuroimaging samples. Overall, this review suggests that PCE is linked to a broad spectrum of detrimental effects and that some biological vulnerabilities associated with PCE may persist. Nonetheless, supportive postnatal environments may mitigate developmental disadvantages and promote better trajectories for affected children. These findings underscore the need for integrated public-health and clinical strategies that combine prevention of prenatal substance use with family-focused postnatal supports.

## 1. Introduction

Cocaine use remains a major and growing public-health concern worldwide [1–4]. The UN Office on Drugs and Crime’s World Drug Report (2025) notes that the number of cocaine users continued to increase, being cocaine the fastest-growing illicit drug market globally [5]. The European Drug Report (2025) similarly highlights that cocaine, after cannabis, is the second most commonly used illicit substance, with rising availability across countries and signals of increasing health and social harms. The report documents a growing burden on treatment services and emergency departments (cocaine is now frequently reported among first-time treatment entrants and is prominent in acute drug-toxicity presentations), as well as upward trends in wastewater indicators in many cities, together suggesting wider geographical and social distribution of cocaine use [6].

Among the many public-health problems associated with cocaine use [7–10], children’s exposure to this substance has received growing attention [11,12]. This exposure may occur through different routes, including intrauterine, breastfeeding, accidental intake, passive inhalation, and intentional administration.

Cocaine use during pregnancy represents a clinically and public-health relevant concern, although the actual prevalence of PCE remains difficult to estimate because of limited national data, variability in ascertainment methods, and underreporting by mothers [13]. In a US national survey from 1992, 1.1% of women under 44 years reported cocaine use during pregnancy; another report found that, among pregnant women reporting any illicit drug use (2.8%), 10% reported cocaine use [13].

In the 1990s, commentary in the lay press and some scientific discussions framed prenatal cocaine exposure (PCE) as producing dramatic and unavoidable developmental sequelae for exposed children [14]; however, assessing long-term consequences of PCE is challenging, particularly with respect to the specificity and generalisability of cocaine-related effects across studies. The limited availability of human cohorts followed into adulthood and the pervasive influence of environmental and co-exposure factors indeed complicated efforts to isolate cocaine-specific effects [15]. Likewise, differences between study populations (such as variations in prenatal care, concurrent substance use, and living environment) have been identified as key reasons why conclusions about neurobehavioral effects remain difficult to generalise [16].

Given these uncertainties, and in light of the increasing global availability of cocaine together with the limited number of cohorts followed into adulthood, a contemporary, systematic synthesis of the human literature on long-term outcomes following children’s exposure to cocaine is timely. The present review therefore aims to provide an updated, developmentally informed overview of human studies that have assessed outcomes from infancy through adolescence and early adulthood, to summarise the domains in which effects have been reported, and to highlight methodological limitations and implications for prevention and intervention.

## 2. Materials and methods

This systematic review was conducted and reported following the Preferred Reporting Items for Systematic Reviews and Meta-Analyses (PRISMA) statement [17]. A protocol was developed prior to study selection to define the research question, eligibility criteria, and data extraction framework (S1 Text); the review was not registered in PROSPERO.

Studies evaluating and reporting long-term effects of children’s exposure to cocaine were searched in the bibliographic databases PubMed (including PubMed Central and Medline), and Scopus, from inception until August 2025. The search terms were related to exposure to cocaine in the paediatric population and its long-term effects. The following search string was used: (children AND exposure AND cocaine AND long-term AND effects). The PubMed search was run using Title/Abstract field tags, while the Scopus search was run using TITLE-ABS-KEY (title, abstract and keywords). All types of studies were searched. Narrative or systematic reviews and meta-analyses, as well as book chapters, editorials, and conference abstracts, were excluded but screened to identify other potential studies to be included. Cross-sectional studies, case series, and case reports were included. After removing duplicates from the different databases, one legal medical doctor (CC) and one PhD student (RM) screened the titles and abstracts of records identified to remove articles that were clearly irrelevant. The full texts of the selected articles were then reviewed by the two authors to define whether they met the inclusion criteria, consisting of the evaluation of long-term effects of children’s exposure to cocaine, whether occurring in utero or postnatally during childhood. For the purposes of eligibility, “long-term effects” were defined as outcomes assessed beyond the immediate neonatal or acute post-exposure period, rather than by applying a predefined minimum age at follow-up. This definition included developmental, cognitive, behavioural, neurobiological, or physical outcomes detectable after the acute phase, including manifestations that persist, emerge, or remain clinically relevant across subsequent developmental stages. Accordingly, studies assessing outcomes in infancy or toddlerhood were considered eligible when they addressed effects not limited to transient neonatal manifestations. In cases of disagreement between the two reviewers at either the title/abstract screening or full-text eligibility stage, discrepancies were resolved through discussion. When consensus could not be reached, a third senior author (CT) acted as an independent adjudicator to resolve conflicts.

Studies conducted on animal models and studies published before 2000 were excluded. The exclusion of studies published before 2000 was intended to focus on more contemporary cohorts with improved exposure assessment methods.

Data from each article included in the systematic review were extracted by the two reviewers. For each article, data on the following items were retrieved: author(s), year of publication, number of children enrolled, age of children, timing of exposure and method of its assessment, exposure to other substances, context, long-term effects at follow-up.

A narrative synthesis approach was adopted, structured by developmental domain (cognitive and neurodevelopmental, behavioural and emotional, neurobiological and functional, and physical growth and health outcomes) and age at assessment.

Risk of bias was assessed using a modified Newcastle–Ottawa Scale, with selected items adapted to better reflect the characteristics of the included studies; the modified tool is provided in S2 Text.

## 3. Results

**Fig 1:**
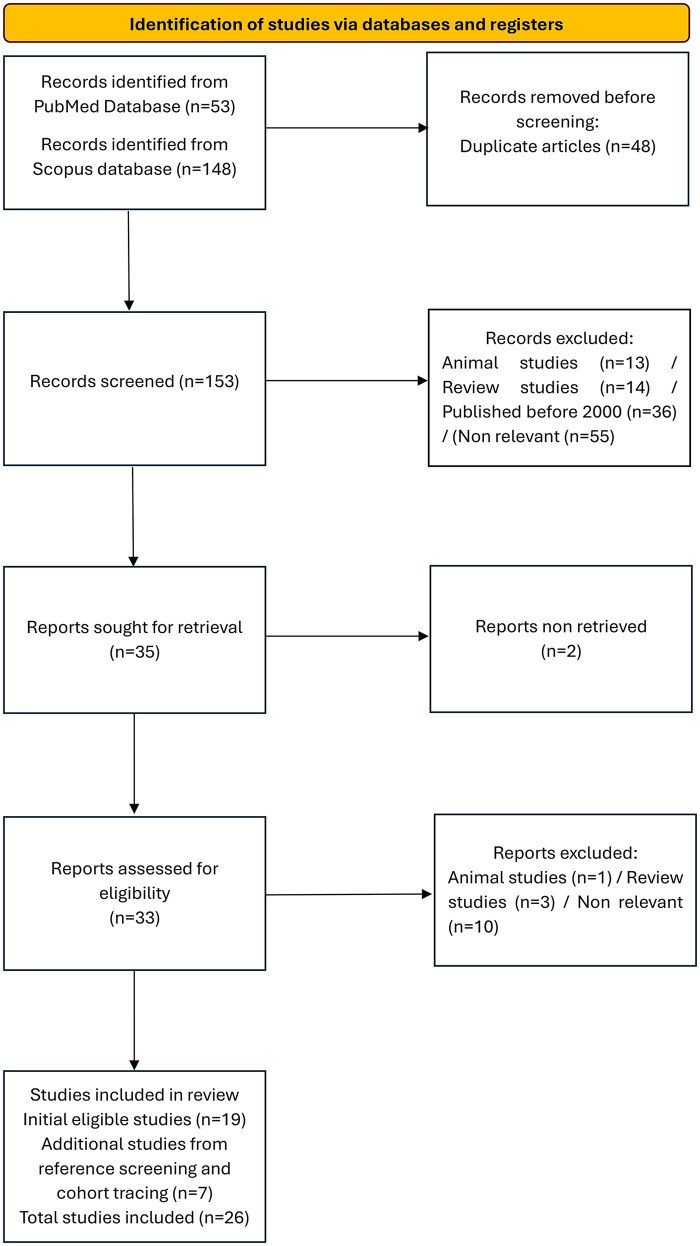
PRISMA flow diagram.

The PRISMA flowchart summarizing the study selection process is shown in Fig 1 [17]. The search strategy allowed the identification of a total of 201 records. After removing duplicates, 153 articles remained for title and abstract screening. Of these, 118 articles were excluded at title-abstract screening, consisting in animal studies (n = 13), review articles (n = 14), published before 2000 (n = 36), or not relevant to the topic (n = 55). Therefore, 35 full-text articles were sought for retrieval; however, 2 reports could not be retrieved, and 33 full-text articles were assessed for eligibility. Of these, further 14 were excluded at full-text reading because they were animal studies (n = 1), review articles (n = 3), or not relevant (n = 10). By screening the reference lists of the 19 studies that met the eligibility criteria, 4 additional eligible studies were identified. Three further studies were included through additional targeted searching, as they referred to longitudinal cohorts already represented or cited in the included articles. Consequently, a total of 26 studies were included in the final review. The characteristics of the included studies are summarized in Table 1.

**Table 1:** Methodological approaches and findings of the reviewed studies.

Article	Sample size	Study Design and Age at Follow-up	Exposure Period	Exposure Assessment	Polysubstance Exposure	Covariates/Contextual Factors	Outcomes at Follow-up
Singer et al. (2024)	384 infants at birth (196 prenatally cocaine-exposed [PCE], 188 non-exposed [NCE]). At the 21-year follow-up, data were available for 325 participants	Prospective Longitudinal Cohort Study (21-year follow-up)	Prenatal (in utero)	- Maternal/infant urine toxicology - Infant meconium analysis - Maternal self-report (Timeline Follow Back)	Alcohol, tobacco, marijuana, (higher in PCE group)	- Low socioeconomic status - Children with NCE had higher lead levels than children with PCE - Analyses controlled for other prenatal substance exposures, HOME score, and in sensitivity models lead exposure; mediation analysis examined birth head circumference and 12-month MDI	A negative association was found between PCE and adult Perceptual Reasoning IQ (PRIQ), mediated by birth head circumference (p = 0.02) and the 12-month Bayley Mental Development Index (MDI) (p = 0.03).
Singer et al. (2023)	325 (163 prenatally cocaine-exposed [PCE], 162 non-exposed [NCE])	Prospective Longitudinal Cohort Study (21-year follow-up)	Prenatal (in utero)	- Maternal/infant urine toxicology - Infant meconium analysis - Maternal self-report (Timeline Follow Back)	Alcohol, tobacco, marijuana, opiates, benzodiazepines (higher in PCE group)	- Low socioeconomic status - Birth mothers in PCE group had fewer years of education, lower marriage rates and marginally higher psychological distress - 32 PCE subjects in non-kin foster/adoptive (FA) care (placed before age 4) - Lower lead levels in PCE/FA group - Higher HOME scores (better environment) in PCE/FA group - Analyses controlled for sex and compared PCE, PCE/foster-adoptive care, and NCE groups using MANCOVA/post-hoc analyses	**Cognitive**: Lower full-scale IQ (83.7 vs. 87.3) and perceptual reasoning (87.3 vs. 91.4) in PCE. **Functional**: Lower high school graduation (75% vs. 86%), marginally higher probation rates. **Behavioral**: No group differences in self-reported substance use disorders. **PCE/FA Subgroup**: Better vocabulary and graduation rates vs. PCE in birth/kinship care.
Powers et al. (2023)	338 adolescents (167 PCE, 171 NCE)	Prospective longitudinal cohort study (17-year follow-up)	Prenatal (in utero)	- Maternal self-report - Maternal/infant urine toxicology - Infant meconium analysis	The PCE group had higher prenatal exposure to alcohol, tobacco, and marijuana	- Low-income, high-risk urban cohort - More foster/adoptive care by age 15 in the PCE group - CE mothers were older, had more children, fewer prenatal visits, lower education, lower PPVT scores, and more psychological distress - PCE infants had shorter gestation and lower birth weight, length, and head circumference - Multiple regression analyses adjusted for HOME score at age 4, sex, birthmother vocabulary (PPVT-R), and child race/ethnicity; PCE × sex interactions were tested	- Adolescents with PCE scored lower on measures of **phonological awareness** (p = .008, ηp² = .030), particularly on the Elision subtest (p = .002, ηp² = .038), and on the reading-related skill pseudoword decoding (p = .023, ηp² = .023). - Girls with PCE scored lower on measures of language memory (p = .047, ηp² = .024), listening comprehension (p = .024, ηp² = .028), and oral discourse comprehension (p = .045, ηp² = .023) compared to girls with NCE.
Kim et al. (2022)	327 (162 prenatally cocaine-exposed [PCE], 165 non-exposed [NCE])	Prospective Longitudinal Cohort Study (17-year follow-up)	Prenatal (in utero)	- Maternal/infant urine toxicology - Infant meconium analysis - Maternal self-report	Alcohol, tobacco, marijuana (higher in PCE group)	- Low socioeconomic status - Mothers of PCE adolescents had less prenatal care, were older and more often unmarried than NCE mothers. They also had poorer vocabulary and higher psychological distress. - Childhood maltreatment (higher in PCE group) - Non-kin foster/adoptive care (higher in PCE group) - Lower gestational age/birth weight in PCE group - Analyses controlled for other prenatal substance exposures, maternal/caregiver factors, HOME score, childhood maltreatment, and adolescent IQ; sex interactions and mediation analyses examining substance use by age 15 were performed	**Mental Health (Self-Reported, CDISC-IV)**: - **Girls with PCE**: 3.6 times higher odds of oppositional defiant disorder (ODD) symptoms vs. NCE girls (p = 0.006). - **Mediation**: Marijuana use by age 15 partially explained ODD symptoms in PCE girls.
De Genna et al. (2022)	193 (75 PCE, 118 non-exposed)	Prospective Longitudinal Cohort Study (25-year follow-up)	First trimester (all PCE cases)	Maternal self-report (interviews on cocaine/crack use by quantity/frequency)	Higher tobacco, alcohol, cannabis, and other illicit drugs in PCE mothers	- Maternal: Older age, single status (PCE > NCE) - Offspring: PCE individuals were more likely to report a history of child abuse, initiate cannabis use by age 15, and have sex at an earlier age - Regression analyses controlled for sex, race, other prenatal substance exposure, family history of alcohol/drug problems, and childhood internalizing problems; indirect pathways were tested with SEM using early cannabis use and age at sexual initiation	PCE individuals were at greater risk of sex under the influence of alcohol or drugs via earlier initiation of cannabis use during adolescence (p = 0.02).
Richardson et al. (2019)	225 (92 PCE, 133 non-exposed)	Prospective Longitudinal Cohort Study (21-year follow-up).	Prenatal (in utero)	Maternal self-report (interviews on cocaine, alcohol, tobacco, marijuana, and other illicit drug use)	Concurrent use of alcohol, tobacco, marijuana, and other drugs (higher in PCE group)	- Mothers in the PCE group were significantly more likely to be older, single, have lower family incomes - Family history of alcohol/drug problems (higher in PCE group) - Higher childhood trauma in PCE group	**Behavioral problems and emotional regulation:** - direct associations between PCE and emotion regulation problems, arrest history, and Conduct Disorder. **Substance Use:** - direct associations between PCE and early initiation of marijuana use (<15 years: 43.5% PCE vs. 18% NCE).
Landi et al. (2017)	110 adolescents (59 PCE, 51 non- exposed [NDE]). ERP subsamples included 70 and 60 participants	Prospective longitudinal cohort study, with follow-up in late adolescence (ages 14–20; mean age: 17.11 years)	Prenatal (in utero)	- Maternal self-report - Maternal urine toxicology - Infant meconium analysis	The PCE group also had higher prenatal exposure to alcohol, tobacco, and marijuana	- Low-SES, urban high-risk cohort - Mothers of PCE adolescents had less education and more prenatal risk factors - More PCE adolescents had a history of foster/adoptive care by age 15 - Analyses controlled for age, grade, SES, other prenatal substance exposure, and nonverbal IQ	- PCE adolescents showed **poorer reading and language performance** overall, with significant deficits in **recalling sentences**, **word/letter reading**, and **reading comprehension** (*p* = .007,.003, and.004; partial η² = .07–.08). - On the ERP tasks, PCE adolescents were less accurate on **rhyme judgments** (*p* = .008) and **sentence anomaly judgments** (*p* < .001). - ERP results showed atypical early phonological processing and **altered N400 responses** in PCE adolescents.
Barthelemy et al. (2016)	140 (69 no Intrauterine Cocaine Exposure [IUCE], 47 lighter IUCE, 24 heavier IUCE)	Prospective Longitudinal Cohort Study (follow-up at 8–11 years)	Prenatal (in utero)	- Maternal self-report - Bioassays of maternal/infant urine and infant meconium - Heavier exposure: Top quartile of self-reported use or top quartile levels of cocaine metabolites in meconium	The sample showed significant differences based on IUCE level (None, Lighter, or Heavier) for intrauterine alcohol, marijuana, and tobacco exposure	- Children from low-income, urban backgrounds − 76% cared for by birth mothers; 24% in non-birth-mother care (relative/foster/adoptive)	- No significant association between IUCE and aggressive behavior. - Positive association between Exposure to violence and aggressive behavior.
Chiriboga et al. (2014)	380 infants (113 cocaine-exposed: 68 CE1 [low], 45 CE2 [high]; 267 unexposed [CU])	Prospective Longitudinal Cohort Study (follow-up at 6, 12, and 24 months)	Prenatal (3rd trimester, quantified via hair segmental analysis)	- Maternal self-report - Biological markers of drug exposure obtained at the time of delivery (hair in all, meconium and urine in a subset). - Dose groups: CU (<5 ng/10 mg hair), CE1 (≤1.5 log ng/10 mg), CE2 (>1.5 log ng/10 mg)	- Higher rates of exposure to tobacco and marijuana in the cocaine exposed infants. - Alcohol: No group differences	- Low socioeconomic status - Maternal: Lower education, unemployment, higher rate of pregnancies in cocaine-using mothers - Neonatal: cocaine-exposed children had lower weight, length and head circumference when compared to unexposed infants - Analyses controlled for HOME score, maternal IQ/depression, welfare status, prematurity, head size, sex, and other prenatal substance exposure; mixed-effects growth-curve models examined microcephaly, birth weight, and hypertonia as potential mediators	- Differences in mental performance over the first 2 years of life associated with prenatal cocaine exposure that was mediated by microcephaly. - Early neurological (hypertonia) and behavioral findings associated with prenatal cocaine exposure improved over time.
Lewis et al. (2013)	364 children (183 prenatally cocaine-exposed [PCE], 181 non-exposed [NCE])	Prospective Longitudinal Cohort Study (12-year follow-up).	Prenatal (in utero)	- Maternal/infant urine toxicology - Infant meconium analysis - Maternal self-report (interviewed at birth about frequency/amount of drug use)	Mothers who used cocaine were more likely to use tobacco, alcohol, and marijuana in greater amounts during pregnancy than were mothers who did not use cocaine.	- Maternal: Lower education, vocabulary (PPVT-R), fewer prenatal visits - Child: Lower birth weight, head circumference, gestational age - Placement: 22.4% of PCE children in foster/adoptive care vs. 4.4% NCE - Environment: Lower HOME scores in PCE with biological/relative care vs. adoptive/foster - Regression analyses controlled for other prenatal substance exposure, maternal/caregiver characteristics, HOME score, and child IQ; subgroup comparisons examined foster/adoptive versus biological-relative care	**Language/Phonology:** - PCE has small, subtle effects on specific aspects of language, including syntax and phonological processing. - **Unexpected:** Better *Rapid Letter Naming* in PCE group (this may be due to the increased impulsivity observed in PCE children). **Environment:** Maternal vocabulary and HOME scores significantly predicted language outcomes.
Lebel et al. (2013)	42 youth (Total: Control = 13, PCE = 12, CAE = 17)	Prospective Longitudinal Cohort Study (follow-up at 14–16 years)	Prenatal (in utero)	- Maternal interview - Unanticipated maternal urine screens (at study enrollment and delivery)	- PCE Group: Cocaine only (no alcohol) - CAE Group: Cocaine and Alcohol - All Groups: Potential exposure to marijuana and tobacco - Tobacco exposure was significantly higher in both cocaine-exposed groups compared to controls	- Postnatal cocaine use: Hair tests at ages 10.5 and 12.5; only one subject (PCE group) tested positive at both time points, suggesting that most positive tests reflect experimental rather than regular use - Analyses tested prenatal tobacco and marijuana exposure as covariates	**Cognitive/Executive Function:** - The CAE group performed significantly worse (slower) on the Trail Making Test B than both other groups (p = 0.006). - No other significant group differences in WCST or Stroop tests were found. **Brain Structure (DTI):** - PCE Group: Lower fractional anisotropy (FA) in right arcuate fasciculus (point-group interaction p = 0.0026); Higher mean diffusivity (MD) in splenium of corpus callosum compared to controls (point-group interaction p = 0.0008). - CAE Group: Higher FA and/or lower MD in right arcuate fasciculus and cingulum compared to other groups. **Structure-Function Relationships:** - Correlations found between diffusion parameters (FA/MD) in affected tracts and performance on executive function tests (Trail Making A/B, Stroop, WCST). These diffusion differences in adolescents with prenatal cocaine exposure suggest localized, long-term structural brain alterations that may underlie attention and response-inhibition difficulties.
Bauer et al. (2011)	725 children (76 high PCE, 170 some PCE, 479 non-exposed)	Prospective Longitudinal Cohort Study (11-year follow-up)	Prenatal (in utero)	- Infant meconium analysis - Maternal self-report	PCE was associated with an increased likelihood of prenatal exposure to alcohol, binge drinking, tobacco, or marijuana	- Children with any cocaine exposure had mothers with less education, had caregivers with higher SES, experienced more caretaker changes, were more likely to have been placed in foster care, and were exposed to more community violence in contrast to children with no PCE - Children with high PCE (vs. nonexposed) were more likely to be born SGA (small for gestational age) - Children with some PCE (vs. nonexposed) were more likely to have caregivers who reported recent alcohol consumption, binge drinking, and greater tobacco use - Analyses controlled for site, birth outcomes, gender, maternal demographics, other prenatal drug exposures, and caretaking environment	Children with higher degrees of PCE exhibited blunted overnight increases in cortisol levels (p = 0.046).
Lewis et al. (2011)	350 children (175 prenatally cocaine-exposed [PCE], 175 non-exposed [NCE])	Prospective Longitudinal Cohort Study (10-year follow-up)	Prenatal (in utero)	- Maternal/infant urine toxicology - Infant meconium analysis - Maternal self-report	Mothers in PCE group used other drugs (i.e., alcohol, marijuana and tobacco) during pregnancy more frequently and in higher amounts than non-users	- Low socioeconomic status - Cocaine using women were older, had more children, and received fewer prenatal care visits than controls - PCE infants were more likely to be preterm and of lower birth weight, head circumference (employed as a mediator of the cocaine effect), and birth length than NCE infants - By 10 years, 39 (22%) PCE children were in adoptive/foster care compared to 7 (4%) of NCE children - Regression analyses controlled for HOME, gender, caregiver vocabulary and psychological symptoms, lead exposure, other prenatal substance exposure, and current placement	- Prenatal cocaine effects were observed for specific aspects of language including syntax (Sentence Combining subtest of the TOLD-I:3, p = 0.001), semantics (Malopropism subtest of the TOLD-I:3, p = 0.05) and phonological processing (Phonological Awareness Subscale of the CTOPP, p = 0.01). - PCE children who experienced foster or adoptive care had enhanced language development compared to those living with birth mothers or in relative care. - Environmental factors (i.e., postnatal lead exposure, home environment, and caregiver vocabulary and psychological symptoms) also impact language skills at 10 years.
Bridgett and Mayes (2011)	284 children (165 prenatally cocaine-exposed [CE], 119 non-cocaine exposed [NCE])	Prospective longitudinal study (inhibitory control was assessed at 7.5, 9.5, and 11.5 years, and aggression was assessed at 14 years)	Prenatal (in utero)	- Maternal self-report - Maternal urine toxicology	The CE group also had high prenatal exposure to alcohol, tobacco, and marijuana	- Low-income, urban high-risk sample - CE children had lower maternal education and more socioeconomic risk - Cumulative risk was defined using low maternal education, low birth weight, and low 2-minute APGAR score - CE children had more adverse birth outcomes than NCE children - Gender and cumulative risk were tested as predictors	**Inhibitory control**: - CE children made more inhibitory control errors at 7.5 years than NCE children (p < .05), but both groups improved over time and CE children showed faster improvement in error rate. - NCE children showed faster improvement in Stroop completion time over development (p < .05), while girls improved faster than boys in both groups. - Higher cumulative risk was associated with poorer inhibitory control performance and slower improvement over time (p < .05). **Aggression outcomes:** - Poorer inhibitory control in childhood predicted higher aggressive behavior at 14 years (p < .05).
Carmody et al. (2011)	203 children (120 unexposed, 38 lightly cocaine-exposed, and 45 heavily cocaine-exposed)	Prospective longitudinal cohort study; assessmentsat 6, 9, and 11 years of age	Prenatal (in utero)	- Maternal self-report - Infant meconium analysis - Cocaine exposure was classified as light or heavy based on frequency of maternal use	The cocaine-exposed group had higher prenatal exposure to alcohol, cigarettes, and marijuana	- High-risk urban sample - Cocaine-exposed children had more neonatal medical complications - Greater environmental risk (including maternal stress, low social support, family instability, low maternal education, and public assistance) in the subgroup of heavily exposed children - Regression analyses controlled for polydrug exposure, medical complications, and environmental risk; analyses were stratified by gender and exposure level	- Prenatal cocaine exposure had a **gender-specific effect**: heavily exposed boys showed poorer overall accuracy in the attention and inhibition task than unexposed or lightly exposed boys (80.95% vs 89.22% and 87.49%, p < .05). - Heavily exposed boys also made more **attention errors** than unexposed boys and lightly exposed boys (25.44% vs 15.73% and 17.71%, p < .02 and p < .05). - Heavily exposed boys showed a trend toward more **inhibitory errors**, although cocaine exposure was not significantly associated with inhibitory errors.
Sheinkopf et al. (2009)	24 children (12 prenatally cocaine-exposed [PCE], 12 non-exposed [NCE])	Prospective Longitudinal Cohort (Point-in-time analysis at 8–9 years)	Prenatal (in utero)	- Meconium toxicology - Maternal self-report	PCE group: 11/12 exposed to alcohol, tobacco, or marijuana NCE group: 7/12 exposed to alcohol/ tobacco	- ADHD status: 3 PCE and 2 NCE children later diagnosed with ADHD by age 11 - Prematurity: may confound temporal lobe effects	**fMRI Task (Go/Nogo Response Inhibition)**: - **Behavioral Performance**: No group differences in accuracy (% correct go/no-go trials). - **Neural Activation**: - PCE Group: Greater activation in right inferior frontal cortex and caudate body during inhibition (p < 0.001). - NCE Group: Greater activation in occipital/fusiform regions (visual processing).
Singer et al. (2008)	371 children (192 cocaine-exposed [CE], 179 non-exposed [NCE])	Prospective longitudinal cohort study (9-year follow-up)	Prenatal (in utero).	- Maternal and infant urine toxicology - Infant meconium analysis - Maternal self-report	The CE group had higher prenatal exposure to alcohol, marijuana, and tobacco	- Low socioeconomic status and high-risk urban sample - CE mothers were older, had more children, fewer prenatal visits, lower education, lower vocabulary, and higher psychological distress CE infants had lower birth weight, length, and head circumference, and were more often preterm - A substantial proportion of CE children were placed in foster/adoptive care; foster/adoptive caregivers had better vocabulary and lower distress, and CE children in foster/adoptive care had lower lead exposure - Analyses controlled for HOME score, maternal/caregiver characteristics, polydrug exposure, blood lead level, iron-deficiency anemia, and foster/adoptive care; sex and race interactions and mediation by birth head circumference were examined	- Prenatal cocaine exposure predicted poorer **perceptual reasoning IQ** (β = −0.16, p = 0.05), and the heavier-exposure group performed worse than the NCE group (p = 0.0207). - The proportion of children with significant perceptual reasoning deficits was higher in the CE group (42% vs 32%, p = 0.047). - Cocaine metabolite levels in meconium were related to lower perceptual reasoning and matrix reasoning scores, suggesting a dose-related effect. - The effect of prenatal cocaine exposure on perceptual reasoning was mediated by smaller head circumference at birth. - School achievement was not significantly affected.
Lewis et al. (2007)	398 children (209 cocaine-exposed [CE] and 189 non-exposed [NCE]).	Prospective longitudinal cohort study, with assessments at 1, 2, 4, and 6 years of age.	Prenatal (in utero)	- Maternal/infant urine toxicology - Infant meconium analysis - Maternal self-report	Higher prenatal exposure to tobacco, alcohol, and marijuana in the CE group	- Low socioeconomic status - CE mothers were older, had more children, and fewer prenatal visits - Lower maternal education, vocabulary, and higher psychological distress in the CE group - CE infants had lower birth weight, length, and head circumference, and were more often preterm - More CE children were placed in foster/adoptive care over time - Analyses controlled for maternal age, race, gender, prenatal tobacco exposure, HOME score, caregiver vocabulary (PPVT-R), parity, SES, marital status, and foster/adoptive care	**Language outcomes:** - Prenatal cocaine exposure had a stable negative effect on language over time; CE children scored approximately 0.15–0.18 SD lower than NCE children on total (p = 0.0385) and expressive language (p = 0.048). - The effect on receptive language was borderline significant (p = 0.058). - Cocaine metabolite levels in meconium were associated with poorer language outcomes at 1 and 2 years. - Tobacco exposure was also linked to poorer receptive language. - Environmental factors mattered: higher HOME scores and better caregiver vocabulary predicted better language outcomes.
Richardson et al. (2007)	224 children (99 first-trimester cocaine-exposed [CE], 125 non-exposed [NE])	Prospective Longitudinal Cohort Study (Longitudinal assessments at 1, 3, 7, and 10 years)	First trimester of pregnancy (with some continuing into later trimesters)	- Maternal self-report	Women who used cocaine during the first trimester were significantly more likely to use tobacco, alcohol, marijuana and other illicit drugs during the first trimester than were the nonusers	- First-trimester users of cocaine were older, less likely to be white, less likely to be married, and had lower family incomes and fewer prenatal care visits - CE Infants were more likely to be premature and low birth weight, and they had decreased gestational age, birth weight, length, and head circumference - Non-maternal custody: 21% CE vs. 6% NE at 10 years - Analyses controlled for maternal height, child gender and race, and first-trimester cigarette use	**Growth Deficits**: - **Cross-sectional**: CE children were smaller at 7 and 10 years (e.g., at 10 years, 1 inch shorter, 12 lbs lighter). No differences at 1/3 years. - **Longitudinal (growth-curve model)**: CE children grew at a slower rate for weight (p = 0.00) and head circumference (p = 0.03).
Arendt et al. (2004)	231 children (101 prenatally cocaine-exposed [PCE], 130 non-exposed [NCE])	Prospective Longitudinal Cohort Study (7-year follow-up)	Prenatal (in utero)	- Medical chart review - Maternal or infant urine toxicology - Clinical interviews with mothers	The PCE group had significantly higher exposure to tobacco, alcohol, marijuana	- Low income status - Biological Mothers of PCE group: Had higher parity, were older, had fewer prenatal visits, lower income, and lower receptive vocabulary (PPVT) scores - Child Custody: 56% of PCE children resided outside maternal care vs. 13% of non-exposed children - Home Environment: PCE children had significantly lower HOME scores at age 7 - Infant Growth: PCE children had significantly lower gestational age, and lower height, weight, and head circumference z-scores at birth - Regression analyses controlled for HOME score, biological/ caregiver vocabulary (PPVT), maternal age, parity, prenatal visits, family income, child age, gender, and polydrug exposure	**Cognitive:** - Significantly lower Verbal IQ (p = .03) and Full Scale IQ (p = .05) in PCE group in bivariate analyses. - In regression models, cocaine exposure status (dichotomous) was not a significant predictor of IQ; the biological mother’s vocabulary (PPVT) and home environment were the strongest predictors. **Visual Motor & Motor Skills:** - Significantly lower Visual Motor Integration (VMI) Motor Coordination standard scores in PCE group (p = .02). - Key Finding on Exposure Level: the amount of cocaine exposure (“rocks per week”) significantly predicted poorer VMI total score (p = .03) and marginally predicted poorer total motor score (p = .08), even after controlling for environmental factors.
Nelson et al. (2004)	143 children at 2 years (70 PCE, 73 NCE); 274 children at 4 years (139 PCE, 135 NCE)	Prospective Longitudinal Cohort Study (follow-up at 2 years and 4 years)	Prenatal (in utero)	- Maternal urine toxicology - Infant meconium analysis - Clinical interview	Higher rates of alcohol, tobacco, and marijuana use in cocaine-exposed mothers	- Low socioeconomic status - Biological Mothers of PCE group: Were older, had higher parity, fewer prenatal visits, greater psychological distress - Infant Health: PCE infants had significantly lower birthweight, birth length, and head circumference - Child Custody: Higher proportion of PCE children in non-maternal care (35% vs 4% at 2y; 57.4% vs 7% at 4y) - Environmental Risks: High rates of elevated blood lead levels (≥10 μg/dL) in both groups - Analyses controlled for cocaine status, gender, maternal IQ, lead, and (in 4-year/cumulative models) HOME score	**Iron Deficiency Anemia (IDA):** - The rate of IDA was significantly higher in the PCE group at 4 years (3.6% vs 0%, p = .026). **Neurodevelopmental Outcomes:** - At 2 years: Exposure to IDA was associated with a significant decrease in the Psychomotor Development Index (PDI) score (−12.9 points, p = .012). - At 4 years: “Peak IDA” (anemic at either 2 or 4 years) was associated with a significant decrease in Full Scale IQ (−8.1 points, p = .047). - Cocaine exposure was not a significant predictor of Full Scale IQ when peak IDA and lead were included in the model. - Elevated blood lead levels independently predicted poorer cognitive outcomes (MDI, Performance, and Full Scale IQ) at both ages.
Singer et al. (2004)	376 children (190 cocaine-exposed, 186 non-exposed) at 4-year follow-up	Prospective Longitudinal Cohort Study (4-year follow-up)	Prenatal (in utero)	- Maternal/infant urine toxicology - Infant meconium analysis - Maternal self-report (frequency/amount of drug use per trimester)	Higher rates of alcohol, tobacco, and marijuana use in cocaine-exposed mothers	- Low income status - Women who used cocaine were older, had more children, and were less likely to have prenatal care. They were less likely to be married or to have completed high school, had lower vocabulary scores, and higher psychological distress scores than women who did not use cocaine. - Cocaine-exposed infants were of lower gestational age, birth weight, head circumference, and length than non exposed infants, and were more likely to be preterm, low birth weight, small for gestational age - Placement: 45% of cocaine-exposed children in non-maternal care (12% adoptive, 10% foster, 23% relatives) vs. 5% of non-exposed - Environment: Cocaine-exposed children in foster/adoptive care had more stimulating home environments (higher HOME scores) and caregivers with better vocabulary scores - Analyses controlled for HOME, maternal age, parity, prenatal visits, maternal education/IQ, marital status, SES, caregiver IQ, psychological distress, foster/ adoptive care, and polydrug exposure	**Cognitive**: - No significant difference in full-scale, verbal, or performance IQ after covariates. - PCE related to deficits in specific subscales: visual-spatial skills (object assembly) (p = 0.01), general knowledge (information) (p = 0.04), and arithmetic (boys only [sex-by-exposure interaction p < 0.03]). - Lower likelihood of IQ > 100 (OR = 0.26). **Environmental Effects**: - Cocaine-exposed children in foster/adoptive care had IQs similar to non-exposed children.
Lewis et al. (2004)	374 children (189 cocaine-exposed [PCE], 185 non-exposed [NCE])	Prospective Longitudinal Cohort Study (4-year follow-up)	Prenatal (in utero)	- Maternal/infant urine toxicology - Infant meconium analysis - Maternal self-report	Higher rates of alcohol, tobacco, and marijuana use in cocaine-exposed mothers	- Low socioeconomic status - Women who used cocaine were older, had more children, and received fewer prenatal care visits - Lower maternal education, vocabulary scores, and higher psychological distress in PCE group - Cocaine-exposed infants had lower gestational age, birth weight, head circumference, and birth length - Higher rates of foster/adoptive placement in PCE children (22% vs. 8% in NCE at 4 years) - Foster/adoptive caregivers had higher HOME scores and better verbal abilities - Regression analyses controlled for HOME, maternal/caregiver IQ and psychological distress, SES, prenatal care, parity, foster/adoptive care, and polydrug exposure; birth parameters and performance IQ were examined as mediators/moderators	**Language outcomes:** - PCE children showed poorer expressive language (p = 0.02) and total language scores (p = 0.04) after controlling for confounders. - Deficits were particularly evident in the Basic Concepts subtest (p = 0.01). - Cocaine-exposed children had more mild receptive language delays (p = 0.039) and were less likely to demonstrate high expressive language abilities (p = 0.014). - Prenatal cigarette and marijuana exposure were associated with deficits in specific language domains. - PCE children placed in foster/adoptive care demonstrated better language outcomes than PCE children remaining in biological/relative care.
Covington et al. (2002)	540 children (231 cocaine-exposed, 309 non-exposed) at 7-year follow-up	Prospective Longitudinal Cohort Study (7 year follow-up)	Prenatal (in utero)	- Maternal self-report - Maternal/infant urine drug screens - Infant meconium analysis	Women who used cocaine during pregnancy used more alcohol and cigarettes during pregnancy than did the remaining mothers	- Mothers who used cocaine during pregnancy were significantly older - The cocaine-exposed children had shorter gestational duration - Placement: Cocaine-exposed children less likely to be in biological maternal care at follow-up - Moderator: Maternal age > 30 amplified growth deficits - Regression analyses controlled for other prenatal exposures, parental height, maternal age/ prepregnancy weight, SES, caretaker characteristics, household composition, maternal psychosocial factors, child lead level, ADHD diagnosis, and hospitalizations	- Prenatal exposure to cocaine predicted birth weight and length. - At age 7, prenatal cocaine exposure was significantly related to height deficits (cocaine exposed children were up to 1 in. shorter and twice as likely to fall below the 10th percentile in height as the control children [p < 0.01])
Thyssen Van Beveren et al. (2000)	100 infants (25 per group): - BC: Non-exposed, biological parents (low SES) - BD: Cocaine-exposed, biological parents (low SES) - AC: Non-exposed, adopted (middle/high SES) - AD: Cocaine-exposed, adopted (middle/high SES).	Prospective Cohort Study (Point-in-time analysis at 12 months)	Prenatal (in utero)	- Neonatal cocaine-positive test (method unspecified) - Maternal self-report	Excluded polydrug use (alcohol, marijuana, etc.) to isolate cocaine effects	- Maternal: Low SES (biological mothers: 75% high school dropouts, 88% single, 89% rental housing) - Adoptive homes: Higher SES (71% college-educated, 87% homeowners, all but one married) - Analyses controlled for gender and ethnicity; adopted/ nonadopted groups were compared to account for postnatal environment	**Cognitive**: - Cocaine-exposed infants (BD/AD) scored lower on: - Spatial Relations (p < 0.0001) - Means-End (p < 0.0001) - Object Permanence (p < 0.04). - No difference in Fagan Test scores. **Motor**: - Fine motor deficits in cocaine-exposed groups (p < 0.0003). - No gross motor differences. **Physical Growth**: - Smaller head circumference (p < 0.0001) and shorter height (p < 0.002) in cocaine-exposed infants. **Postnatal Environment**: - Adoption improved cognitive scores and fine motor skills in non-exposed infants (AC). - No improvement in cocaine-exposed adopted infants (AD) vs. BD.
Delaney-Black et al. (2000)	471 children (201 prenatally cocaine-exposed [PCE], 270 non-exposed controls)	Prospective Longitudinal Cohort Study (6-year follow-up)	Prenatal (in utero)	- Maternal self-report - Maternal/infant urine drug screens - Infant meconium analysis (20% of subjects)	Cocaine-using mothers consumed more alcohol, cigarettes, and marijuana during the index pregnancy	- Cocaine-using mothers were older - The cocaine-exposed children were smaller at birth and had shorter gestational duration - At the 6-year follow-up, exposed children were less likely to be in the care of their mother - Regression analyses controlled for gender, polydrug exposure, child age, SES, caregiver marital status, current drug use in the home, violence exposure, lead level, and custody status	- **Behavioral**: Higher Externalizing-Internalizing Difference scores in PCE group (p = 0.018). - **Gender effects**: PCE boys had 2x higher rates of clinically significant externalizing (25% vs. 13%) and delinquent behaviors (22% vs. 11%). - **Custody changes**: Associated with worse Teacher’s Report Form scores (Total, Externalizing) in PCE group.

All included articles dealt with cocaine exposure that occurred in utero. The results are presented according to developmental domains, with attention to the age at which outcomes were assessed.

### 3.1 Cognitive and neurodevelopmental outcomes

Sixteen of the included studies reported cognitive and neurodevelopmental effects associated with PCE [16,18–32]. In infancy and toddlerhood, differences in mental performance over the first 2 years of life were found to be mediated by microcephaly, while early neurological signs such as hypertonia tended to improve over time [21]. At 12 months, cocaine-exposed infants showed lower scores in spatial relations (p < 0.0001), means–end problem-solving (p < 0.0001), object permanence (p < 0.04), and fine motor skills (p < 0.0003), together with reduced head circumference (p < 0.0001) [32].

At preschool and school age, findings continued to suggest domain-specific cognitive effects. At 4 years, PCE predicted poorer visual–spatial skills (p = 0.01), general knowledge (p = 0.04), and arithmetic among boys (sex-by-exposure interaction p < 0.03), although children in foster/adoptive care had IQ scores similar to non-exposed children [29]. At age 7, lower verbal IQ and full-scale IQ were observed in bivariate analyses, but maternal vocabulary and the home environment, rather than PCE itself, predicted IQ in adjusted models; however, higher levels of cocaine exposure were associated with poorer motor coordination and visual–motor integration (VMI motor coordination p = 0.02; VMI total score predicted by exposure amount, p = 0.03) [18]. At age 9, poorer perceptual reasoning IQ was also reported (p < .05), with a linear relationship between cocaine metabolite levels and degree of impairment and mediation through birth head circumference [30].

Language outcomes showed a relatively consistent pattern of subtle but persistent vulnerabilities from childhood into adolescence. Early language differences included poorer expressive language (p = 0.02) and total language scores (p = 0.04), with deficits particularly evident in the Basic Concepts subtest (p = 0.01), as well as mild receptive language delays (p = 0.039), with better outcomes among children placed in foster/adoptive care [24]. Longitudinally, PCE showed a stable negative effect on total language (p = 0.0385) and expressive language (p = 0.048), while higher HOME scores and better caregiver vocabulary were associated with better language outcomes [25]. At later follow-ups, impairments were reported in syntax (p = 0.001), semantics, and phonological processing (p = 0.01) at age 10, and small but specific language deficits persisted at age 12, although maternal vocabulary and home environment were stronger predictors of performance than PCE per se [26,27]. In adolescence, poorer reading and language performance were reported in recalling sentences, word/letter reading, and reading comprehension (p = .007,.003, and.004), while lower phonological awareness (p = .008), poorer reading-related skills, and additional language deficits in girls were also observed [22,28].

Executive-function findings were also selective. In childhood, PCE was associated with more inhibitory control errors at 7.5 years (p < .05), although both exposed and unexposed children improved over time [19]. Heavily exposed boys showed poorer overall accuracy and more attention errors in attention/inhibition tasks (p < .05), suggesting a possible gender-specific effect of PCE on executive functioning [20]. In adolescence, youth exposed to both cocaine and alcohol performed worse on executive-function tasks, particularly Trail Making Test B (p = 0.006), and adolescents with PCE showed alterations in white matter microstructure, including lower fractional anisotropy in the right arcuate fasciculus (p = 0.0026) and higher mean diffusivity in the splenium (p = 0.0008). These structural differences were correlated with executive-function performance [23].

At the transition to adulthood, evidence from longitudinal follow-up suggested that some selective cognitive and functional differences may persist. At age 21, PCE was negatively associated with perceptual reasoning IQ, with this association mediated by birth head circumference (p = 0.02) and 12-month developmental scores (p = 0.03) [31]. In a parallel analysis of the same cohort, exposed participants showed lower full-scale IQ (83.7 vs. 87.3), lower perceptual reasoning scores (87.3 vs. 91.4), and lower odds of high school graduation (75% vs. 86%) [16].

### 3.2 Behavioural and emotional outcomes

Behavioural effects of PCE varied across development, as indicated by five studies [14,33–36].

In childhood, behavioural findings mainly concerned externalizing and aggressive behaviours, although results were not uniform. At age 6, exposed children displayed higher externalizing behaviours (p = 0.018), with PCE boys showing approximately twice the rates of clinically significant externalizing and delinquent symptoms; custody changes were also associated with worse behavioural outcomes [14]. However, in children assessed between ages 8 and 11, intrauterine cocaine exposure was not associated with aggressive behaviour, whereas exposure to violence emerged as the main predictor of aggression [33].

During adolescence and young adulthood, behavioural outcomes suggested more complex pathways involving sex-specific effects, emotional regulation, conduct problems, and early substance use. At 17 years, girls with PCE had 3.6-fold higher odds of oppositional defiant disorder symptoms (p = 0.006), partly mediated by marijuana use by age 15 [35]. By young adulthood, PCE was associated with emotion-regulation difficulties, arrest history, conduct disorder, and earlier initiation of marijuana use before age 15 [36]. In adulthood, PCE was also associated with having sex under the influence of alcohol or substances, with this pathway mediated by earlier adolescent cannabis initiation (p = 0.02) [34].

### 3.3 Neurobiological and functional outcomes

A small number of studies employed neurobiological [22,23,37] or physiological measures [38]. In children aged 8–9 years, PCE was associated with greater activation of the right inferior frontal cortex and caudate during response-inhibition tasks (p < 0.001), despite comparable behavioural accuracy [37]. Electrophysiological evidence suggested atypical language-related processing, with older adolescents with PCE showing atypical N400 responses [22].

Physiological stress regulation was examined at age 11, with higher levels of PCE associated with blunted overnight increases in cortisol (p = 0.046) [38].

### 3.4 Physical growth and health outcomes

Four studies observed growth and health effects across infancy, childhood, and preadolescence [32,39–41].

Across studies, PCE was associated with reduced growth parameters, particularly head circumference, height, and weight. In infancy, exposed children showed smaller head circumference (p < 0.0001) and shorter height (p < 0.002) [32]. Growth differences were also observed later in childhood: children exposed in the first trimester were smaller at ages 7 and 10 and showed slower growth in weight (p = 0.00) and head circumference (p = 0.03) over time [41]. At age 7, PCE predicted height deficits, with exposed children being up to one inch shorter and twice as likely to fall below the 10th percentile for height (p < 0.01) [39].

Health-related findings indicated that, by age 4, iron-deficiency anemia was more common in the PCE group (p = 0.026). Iron-deficiency anemia at 2 years was strongly associated with poorer motor scores (p = 0.012), and anemia at 2 or 4 years was associated with poorer total IQ. Once iron-deficiency anemia and lead levels were included in the model, PCE was no longer a significant predictor of IQ [40].

### 3.5 Environmental factors and polysubstance exposure

Across studies, concurrent exposure to alcohol, tobacco, and marijuana was common among children of cocaine-using mothers. Multiple cohorts, including those reported by Singer et al. [16,29], Lewis et al. [26,27], Arendt et al. [18], and Nelson et al. [40], highlighted the critical role of postnatal environment: foster or adoptive care, higher HOME scores, and higher caregiver vocabulary consistently predicted better cognitive outcomes in children with PCE. In contrast, exposure to community violence, child maltreatment, caregiver instability, and other environmental adversities were examined as potential contributors to developmental risk.

Because PCE rarely occurred in isolation, studies commonly adjusted for other prenatal substance exposures, SES, HOME score, caregiver characteristics, maternal psychological distress, foster/adoptive care or custody changes, lead exposure, and relevant medical or perinatal risks. Some studies also examined interactions or stratified effects by sex, exposure level, maternal age, or placement/caregiver status [16,20,24,27–30,35,39], and a smaller number tested mediation pathways involving birth head circumference, early developmental indicators, later substance use, or caregiving environment [21,30,31,34,35]. Overall, the included studies were consistent in reporting adjusted associations and, in some cases, mediation or moderation pathways, but none reported definitive causal relationships.

### 3.6 Risk of bias assessment

Using the modified Newcastle-Ottawa scale, none of the included studies was judged to be at high risk of bias; 7 studies were rated as having a moderate risk of bias, and 19 studies were rated as having a low risk of bias (S1 Table).

## 4. Discussion

This review aimed to explore evidence on long-term effects of children’s exposure to cocaine. By drawing exclusively on human cohorts followed from infancy into childhood, adolescence and early adulthood, the review aims to provide a focused, developmentally framed picture of the range of cognitive, behavioural, neurobiological and health outcomes reported in the literature. Interestingly, all studies meeting selection criteria and included in this review refer to exposures that occurred in utero.

Unlike previous reviews examining a broad range of substances (e.g., alcohol, nicotine) [42,43] or focusing on prenatal cocaine effects on a specific age (e.g., childhood) [44,45], this review examines the long-term effects of PCE across a wide developmental span, from infancy to young adulthood. This lifespan perspective allows for a more comprehensive understanding of the risks associated with in utero cocaine exposure. Moreover, whereas some prior syntheses have relied on animal models [46,47], this review draws exclusively on human research to characterise the nuanced developmental sequelae of PCE. By adopting this human-focused lens, the analysis provides insights directly applicable to real-world populations.

The reviewed studies collectively suggest that prenatal exposure to cocaine is associated with a broad spectrum of effects across multiple developmental domains. These sequelae are generally described as modest and domain specific. Early neurodevelopmental impairments, along with growth deficits, have been documented in infancy and toddlerhood [21,32], while later cognitive and behavioural sequelae tend to be focal (perceptual reasoning, specific language and phonological skills, executive/attention networks, and elevated externalizing behaviours). Specific language and phonological deficits have been reported from childhood to adolescence [22,24–28], while impairments in perceptual reasoning have been observed in middle childhood into young adulthood [16,30,31]. Behavioural elevations, particularly in externalizing problems, were described in several cohorts [14,35,36]. Neurobiological studies (although based on relatively small samples) point to focal alterations in white-matter microstructure and functional recruitment during tasks of inhibition and attention [23,37], along with atypical ERP responses during language processing [22]. Overall, these converging findings support the possibility of persistent adverse effects of prenatal cocaine exposure on brain development [21].

A recurring and central theme across the literature is that PCE rarely occurs in isolation. Concurrent prenatal exposure to alcohol, tobacco and marijuana was common across cohorts, and most samples were drawn from low-income, inner-city populations. For example, Arendt et al. [18] observed that both exposed and unexposed children from disadvantaged backgrounds often perform below expectations, and poverty-related risks can produce progressive developmental lags that may rival or exceed the impact of prenatal drug exposure. Socioeconomic adversity and polysubstance exposure therefore appear important contextual determinants that complicate causal attribution and magnify risk.

The pattern of results (consistent perinatal effects, selective cognitive deficits, focal neurobiological changes, and influence of postnatal context) suggests a multifactorial model. Biological effects of PCE (e.g., on fetal growth and brain development) are supported by mediational findings in some cohorts, but post-natal environmental risks may attenuate or explain observed associations. Thus, causal interpretation should be cautious: some impairments may be directly related to prenatal cocaine’s teratogenic potential, while others reflect the combined influence of prenatal polysubstance exposure and adverse postnatal environments.

Importantly, multiple cohorts highlighted that postnatal environment matters for later outcomes. According to Singer et al. [16], while some cognitive differences may have a biological basis, functional outcomes are modifiable through environmental interventions. Similarly, as Arendt [18] reported, although PCE may confer vulnerability in specific domains (for example, visual–motor skills), inadequate rearing environments are often stronger predictors of children’s developmental trajectories than prenatal exposure alone. Across studies, these observations are supported by evidence that foster/adoptive care, higher HOME scores, and higher caregiver vocabulary or IQ were associated with better cognitive or language outcomes among children with PCE, whereas violence exposure, lead exposure, and other environmental risks were associated with poorer outcomes [16,18,24–27,29,40]. These findings suggest an important, positive implication: targeted postnatal supports and interventions may reduce functional disadvantage and promote resilience. The reviewed evidence thus supports dual-action strategies that combine prenatal substance-exposure prevention with postnatal family- and environment-focused interventions.

## 5. Limitations and future perspectives

Several limitations of the primary literature constrain definitive conclusions. Heterogeneity across cohorts (exposure assessment methods, covariate control, outcome measures, ages at follow-up) is substantial; some reviewed studies relied primarily on maternal self-report or on maternal/infant urine toxicology to identify exposed children, whereas other studies were based on meconium testing, and only one employed hair analysis. In this regard, it should be noted that hair analysis is a very effective method for drug-use investigation and offers a long detection window, but the finding of cocaine in the hair of infants should be interpreted cautiously: infant hair is thinner and more porous and therefore more prone to external contamination, and in the first months of life it can be difficult to disentangle in utero incorporation from postnatal exposures [11,48]. This further highlights the challenges in accurately characterising exposure in the reviewed cohorts. Sample sizes for neuroimaging studies are small; moreover, polysubstance use and socioeconomic confounding remain difficult to fully disentangle. Given this substantial heterogeneity across studies, a narrative synthesis without quantitative meta-analysis was performed; consequently, pooled effect estimates could not be generated, limiting the ability to precisely quantify effect sizes. Finally, publication bias and the focus on low-SES cohorts may reduce generalisability to other populations.

Future research should prioritise large, well-characterised longitudinal cohorts with rigorous, multi-method exposure assessment, better control for co-exposures and socioeconomic confounders, and sufficiently powered neuroimaging and intervention studies to test causality and plasticity. Evaluations of postnatal interventions would be particularly valuable to determine which strategies most effectively affect outcomes.

## 6. Conclusions

This review suggests, on the one hand, that prenatal cocaine exposure is associated with cognitive, behavioural and neurobiological effects, and, on the other, that supportive postnatal environments may mitigate many functional outcomes. In particular, higher-quality home environments and higher caregiver IQ or vocabulary were associated with better cognitive or language outcomes among children with PCE, whereas violence exposure and other environmental risks were associated with poorer outcomes. Considering the observed importance of the postnatal environment, the reviewed evidence supports the implementation of dual-action strategies combining prevention of prenatal substance use with postnatal supports, including coordinated medical monitoring of infant health and growth, maternal substance-use treatment when needed, caregiver support, and early developmental services [13]. Thus, while some biological vulnerabilities may persist, clinical and public-health responses should focus on reducing modifiable environmental risks and promoting stable, enriched developmental contexts for children with PCE.

## Supporting information

S1 Text Study protocol. (DOCX)

S2 Text Adapted Newcastle–Ottawa scale. (DOC)

S1 Table Risk of bias assessments of included studies. (DOCX)

S2 Table PRISMA checklist. (DOCX)
