# Supplementary material for: Long-term effects following prenatal cocaine exposure: A systematic review
Source: PLoS One. 2026 Jun 26;21(6):e0352587. doi: 10.1371/journal.pone.0352587 (PMC13308802; doi:10.1371/journal.pone.0352587)
Supplement: S1 Text — (DOCX) [file pone.0352587.s001.docx]

**S1 Text. Study protocol.**

**Long-term effects of children’s exposure to cocaine**

**Background**

Cocaine a well-known and widespread drug of abuse, to which also children can be exposed. Children exposure to this substance may occur through different routes, including intrauterine, breastfeeding, accidental intake, passive inhalation, and intentional administration. Assessing long-term consequences of exposures occurred in utero and during early infancy is challenging due to the limited availability of human cohorts followed into adulthood and the influence of environmental and co-exposure factors which complicated efforts to isolate cocaine-specific effects.

**Review objectives**

What long-term outcomes are associated with children cocaine exposure?

**Databases**

The literature search is conducted in:

- PubMed (including PubMed Central and MEDLINE)
- Scopus

**PECO approach**

**Population (P)**
Human subjects exposed to cocaine in utero or during childhood.

**Exposure (E)**
Cocaine exposure assessed via maternal self-report (in-utero exposures), clinical records, or toxicological analysis (e.g., meconium, urine, hair).

**Comparator (C)**
Subjects without cocaine exposure during childhood, when comparison groups are available.

**Outcomes (O)**
Any long-term outcomes of children’s exposure to cocaine.

**Eligibility Criteria**

**Inclusion Criteria**

Studies are included if they:

- Involve human participants known to be exposed to cocaine in utero or during childhood.
- Report long-term outcomes of children’s exposure to cocaine.

**Exclusion Criteria**

Studies are excluded if they:

- Are animal studies.
- Are reviews, meta-analyses, editorials, commentaries, or book chapters.
- Were published before 2000.

Reference lists of relevant reviews are screened to identify additional eligible studies.

**Data Extraction**

Data are extracted by two reviewers.

The following information are collected:

- Author(s) and year of publication.
- Study design.
- Sample size.
- Timing of exposure and method of its assessment.
- Exposure to other substances.
- Contextual factors.
- Age at outcome assessment.
- Long-term effects.

**Strategy for data synthesis**

Narrative synthesis of included studies.
