# Supplementary material for: Long-term effects following prenatal cocaine exposure: A systematic review
Source: PLoS One. 2026 Jun 26;21(6):e0352587. doi: 10.1371/journal.pone.0352587 (PMC13308802; doi:10.1371/journal.pone.0352587)
Supplement: S2 Text — (DOC) [file pone.0352587.s002.doc]

**S2 Text. Adapted Newcastle–Ottawa Scale.**

**ADAPTED** **NEWCASTLE - OTTAWA QUALITY ASSESSMENT SCALE**

**COHORT STUDIES**

**Selection**

1) Representativeness of the exposed cohort

a) truly representative of the community (3 points)

b) somewhat representative of the population at risk of being exposed (2 points)

c) selected group of volunteers (1 point)

d) no description of the derivation of the cohort (0 points)

2) Selection of the non exposed cohort

a) drawn from the same community as the exposed cohort (2 points)

b) drawn from a different source (1 point)

c) no description of the derivation of the non exposed cohort (0 points)

3) Ascertainment of exposure

a) child toxicological analysis (3 points)

b) some cases with maternal or infant toxicological analysis, some with maternal self-report (2 points)

c) only maternal self-report (no toxicological analysis) (1 point)

d) no description (0 points)

4) Demonstration that outcome of interest was not present at start of study

a) yes (1 point)

b) no (0 points)

**Comparability**

1) Comparability of cohorts on the basis of the design or analysis

a) study efforts to control for confounding factors (environment / polydrug exposure) (1 point)

b) study do not consider for potential confounders (0 points)

**Outcome**

1) Assessment of outcome

a) independent blind assessment or reference to secure records (3 points)

b) record linkage (2 points)

c) self report (1 point)

d) no description (0 points)

2) Was follow-up long enough for outcomes to occur

a) yes (1 point)

b) no (0 points)

3) Adequacy of follow up of cohorts

a) complete follow up - all subjects (3 points)

b) subjects lost to follow up unlikely to introduce bias - small number lost (2 points)

c) follow up rate < 70% and no description of those lost (1 point)

d) no statement (0 points)

Overall risk of bias was categorised according to predefined thresholds based on the total score as follows: high risk (0–6 points), moderate risk (7–12 points), and low risk (13–17 points).
