# Supplementary material for: Long-term effects following prenatal cocaine exposure: A systematic review
Source: PLoS One. 2026 Jun 26;21(6):e0352587. doi: 10.1371/journal.pone.0352587 (PMC13308802; doi:10.1371/journal.pone.0352587)
Supplement: S1 Table — (DOCX) [file pone.0352587.s003.docx]

**S3 Table. Risk of bias assessments of included studies.**

| **Study** | **Selection** | **Comparability** | **Outcome** | **Total NOS score** | **Overall risk of bias** |
| --- | --- | --- | --- | --- | --- |
| Singer et al. (2024) | 7/9 | 1/1 | 6/7 | 14/17 | Low |
| Singer et al. (2023) | 7/9 | 1/1 | 6/7 | 14/17 | Low |
| Powers et al. (2023) | 7/9 | 1/1 | 6/7 | 14/17 | Low |
| Kim et al. (2022) | 7/9 | 1/1 | 4/7 | 12/17 | Moderate |
| De Genna et al. (2022) | 7/9 | 1/1 | 3/7 | 11/17 | Moderate |
| Richardson et al. (2019) | 7/9 | 1/1 | 4/7 | 12/17 | Moderate |
| Landi et al. (2017) | 7/9 | 1/1 | 6/7 | 14/17 | Low |
| Barthelemy et al. (2016) | 7/9 | 1/1 | 3/7 | 11/17 | Moderate |
| Chiriboga et al. (2014) | 8/9 | 1/1 | 5/7 | 14/17 | Low |
| Lewis et al. (2013) | 7/9 | 1/1 | 6/7 | 14/17 | Low |
| Lebel et al. (2013) | 7/9 | 1/1 | 6/7 | 14/17 | Low |
| Bauer et al. (2011) | 7/9 | 1/1 | 6/7 | 14/17 | Low |
| Bridgett & Mayes (2011) | 7/9 | 1/1 | 3/7 | 11/17 | Moderate |
| Carmody et al. (2011) | 8/9 | 1/1 | 6/7 | 15/17 | Low |
| Lewis et al. (2011) | 7/9 | 1/1 | 6/7 | 14/17 | Low |
| Sheinkopf et al. (2009) | 7/9 | 0/1 | 5/7 | 12/17 | Moderate |
| Singer et al. (2008) | 7/9 | 1/1 | 6/7 | 14/17 | Low |
| Lewis et al. (2007) | 7/9 | 1/1 | 6/7 | 14/17 | Low |
| Richardson et al. (2007) | 7/9 | 1/1 | 6/7 | 14/17 | Low |
| Arendt et al. (2004) | 7/9 | 1/1 | 6/7 | 14/17 | Low |
| Lewis et al. (2004) | 7/9 | 1/1 | 6/7 | 14/17 | Low |
| Nelson et al. (2004) | 7/9 | 1/1 | 5/7 | 13/17 | Low |
| Singer et al. (2004) | 7/9 | 1/1 | 6/7 | 14/17 | Low |
| Covington et al. (2002) | 7/9 | 1/1 | 5/7 | 13/17 | Low |
| Thyssen Van Beveren et al. (2000) | 8/9 | 1/1 | 5/7 | 14/17 | Low |
| Delaney-Black et al. (2000) | 7/9 | 1/1 | 4/7 | 12/17 | Moderate |
